# A Narrative Review of Current and Emerging Trends in the Treatment of Alcohol Use Disorder

**DOI:** 10.3390/brainsci14030294

**Published:** 2024-03-20

**Authors:** Muhammet Celik, Mark S. Gold, Brian Fuehrlein

**Affiliations:** 1 New York Medical College at Saint Joseph’s Medical Center, Yonkers, NY 10705, USA; drcelikmuhammet@gmail.com; 2 Department of Psychiatry, School of Medicine, Washington University, St. Louis, MO 63130, USA; 3 Mental Health Service Line, VA Connecticut Healthcare System, West Haven, CT 06516, USA; brian.fuehrlein@va.gov; 4 Department of Psychiatry, Yale School of Medicine, New Haven, CT 06511, USA

**Keywords:** alcohol, alcohol use disorder (AUD), withdrawal

## Abstract

Alcohol use disorder (AUD) is a significant contributor to morbidity and mortality in the United States. It contributes to over 140,000 annual deaths, to over 200 related diseases and health conditions globally, and accounts for 5.1% of the global disease burden. Despite its substantial impact, AUD remains undertreated, marked by a scarcity of approved medications. This paper explores the current treatment landscape and novel strategies for both alcohol withdrawal syndrome and AUD. Promising results, including the use of psychedelics alongside psychotherapy, noninvasive neural-circuit-based interventions, phosphodiesterase-4 inhibitors, and GLP-1 receptor agonists, have emerged from recent studies. While these advancements show potential, further research is crucial for a comprehensive understanding of their effectiveness. The clear shortage of approved medications and other treatment modalities underscores the pressing need for ongoing research.

## 1. Introduction

Alcohol use is a serious worldwide problem. According to the 2021 National Survey on Drug Use and Health (NSDUH), 78.3% of Americans over the age of 12 have consumed alcohol in their lifetime [1] and approximately 30 million meet the criteria for alcohol use disorder (AUD) [2]. The *Diagnostic and Statistical Manual of Mental Disorders, Fifth Edition, Text Revision* (DSM-5-TR) [2] defines AUD as a problematic drinking pattern causing significant impairment or distress, as indicated by meeting at least 2 of 11 criteria within a 12-month span. These criteria include consuming alcohol in larger amounts or for longer durations than intended, unsuccessful attempts to cut down or control alcohol intake, dedicating substantial time to obtain, use, or recover from alcohol, experiencing strong cravings for alcohol, failing to fulfill major obligations due to alcohol use, persistent social or interpersonal problems resulting from alcohol use, giving up important activities due to alcohol, engaging in alcohol use in physically hazardous situations, continuing alcohol use despite knowledge of related physical or psychological problems, developing tolerance requiring increased alcohol consumption for desired effects, and experiencing withdrawal symptoms when alcohol use is discontinued.

AUD is thus the fourth leading preventable cause of death [3]. Furthermore, alcohol use is believed to contribute to over 200 diseases and health conditions, including cardiovascular disease, cancer, liver cirrhosis, and injuries [4]. Despite being a significant public health concern, AUD is notably undertreated, with only 7% of adults in the US receiving adequate treatment for the disorder [5]. Furthermore, only 16% of individuals undergoing treatment for AUD achieve abstinence, defined as individuals with prior-to-past-year AUD who were in remission within the past year according to the DSM-5-TR remission criteria [6].

Alcohol exerts its influence on the central nervous system (CNS) by impacting various neuromodulators and cellular receptors. It enhances the inhibitory effects of gamma-aminobutyric acid (GABA) while concurrently inhibiting N-methyl-D-aspartate (NMDA) receptors. In the absence of alcohol, this leads to an increased stimulation of glutamate, ultimately resulting in a state of neural excitability [7]. Prolonged ethanol consumption induces a reduction in endogenous GABA release, a down-regulation of GABA-A receptors, an up-regulation of NMDA receptors, and an increase in glutamate production. This results in heightened inhibitory neurotransmitter activity, leading to the development of physical dependence for maintaining equilibrium. Abrupt cessation of alcohol triggers a net excitatory state, characterized by the clinical signs and symptoms of alcohol withdrawal syndrome (AWS) [8].

**Figure 1:**
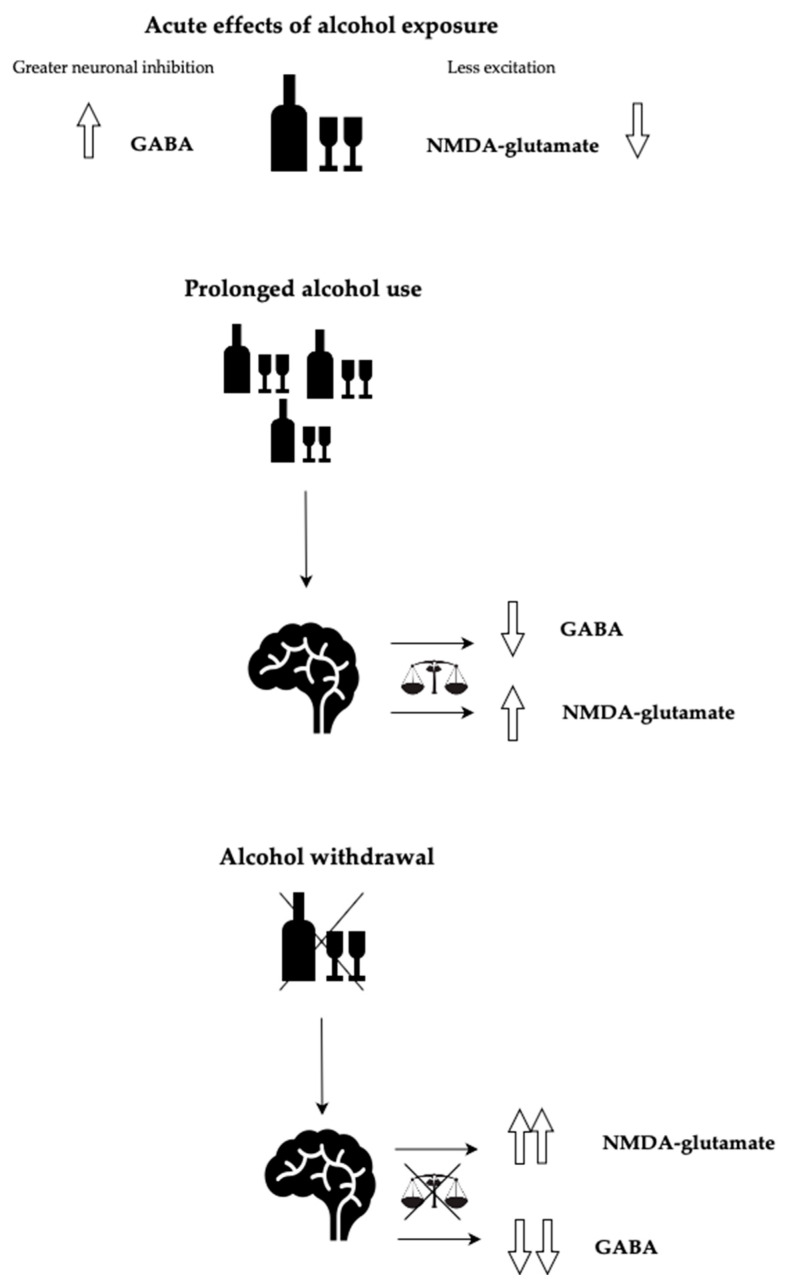
Alcohol’s effects on neurotransmitters and CNS.

Please refer to Figure 1 for a simplified demonstration of alcohol’s effects on neurotransmitters and CNS.

The practice guidelines of the American Psychiatric Association (APA) recommends pharmacotherapy in conjunction with psychosocial interventions for patients with moderate or severe AUD [9]. However, only three medications—disulfiram, acamprosate, and naltrexone—are approved by the US Food and Drug Administration (FDA) for treatment of AUD. Benzodiazepines are currently the first-line treatment for AWS [10]. However, benzodiazepines have adverse effects such as sedation, falls, aspiration, and respiratory depression [11].

Approximately 50% of individuals in the US with AUD have experienced AWS. This percentage rises to over 80% among individuals with AUD who are hospitalized or homeless [2]. According to the DSM-5-TR [2], alcohol withdrawal diagnosis is determined when a person who has engaged in heavy and extended alcohol consumption experiences a minimum of two of the subsequent symptoms within a short timeframe of reducing or stopping alcohol intake: heightened autonomic activity, amplified hand tremors, difficulty sleeping, feelings of nausea or vomiting, hallucinations, increased psychomotor agitation, heightened anxiety, or seizures. These symptoms must result in notable distress or hinder the individual’s ability to function and cannot be ascribed to any other medical or psychological condition. Alcohol withdrawal symptoms typically begin within 6–24 h after the last drink and can vary widely, ranging from mild tremors to severe manifestations such as delirium tremens (DTs).

This review provides an overview of current treatment strategies for AWS and AUD, and explores alternative and novel approaches with promising results, along with recent developments in the treatment of these conditions.

## 2. Methods

This paper presents findings obtained from an electronic literature search across databases, including PubMed, Google Scholar, Cochrane Library, and ClinicalTrials.gov. The search aimed to explore recent advancements, novel pharmacological compounds, and forthcoming research publications for AWS and AUD. Key terms such as alcohol, alcohol use disorder, alcohol withdrawal syndrome, alcohol dependence, alcohol consumption, relapse, and anti-craving were employed in conjunction with various treatment approaches. Our paper provides brief historical data and background information on each treatment modality to offer a broader perspective and focuses on clinical and animal trials, as well as relevant systematic reviews, meta-analyses, and case reports published between 1 January 2013 and 1 January 2024. Editorials, expert opinions, and meeting abstracts were excluded.

## 3. Results

### 3.1. Benzodiazepines for AWS

The primary treatment for AWS is benzodiazepines. There is no specific preference for a particular benzodiazepine in the existing literature, highlighting the need for personalized medication selection, particularly considering factors like liver dysfunction [10]. When liver dysfunction is a concern, lorazepam or oxazepam are the recommended choices, while diazepam can also be utilized due to its prolonged half-life and rapid onset of action. Various dosing strategies, including front loading, fixed-schedule regimens, and symptom-triggered regimens based on The Clinical Institute Withdrawal Assessment Alcohol Scale Revised (CIWA-Ar), are available.

Front-loading is an approach which entails administering high doses of long-acting benzodiazepines, particularly for patients experiencing severe withdrawal symptoms. While this method may offer benefits, there is a risk of oversedation and respiratory depression, necessitating vigilant monitoring. Alternatively, in cases of high-risk alcohol withdrawal or a history of seizures or DTs, a fixed-schedule regimen involving diazepam and chlordiazepoxide may be employed. Another option is a symptom-triggered regimen, where the CIWA-Ar scale is utilized to monitor symptoms, and medications such as diazepam, lorazepam, or chlordiazepoxide are administered as required [12,13]. However, this approach demands close nursing supervision for monitoring and may not be suitable for resource-limited or high-volume settings. Moreover, for patients with a history of complicated withdrawals involving seizures, this strategy may pose safety concerns. With this method, the treatment duration is typically shorter, and lower doses of benzodiazepines may suffice.

### 3.2. Medications Other than Benzodiazepines for Alcohol Withdrawal Syndrome

#### 3.2.1. GABA-B Receptor Agonists

##### Baclofen

Baclofen’s mechanism of action involves the activation of the GABA-B receptor, leading to the down-regulation of GABA-A activity. This process establishes a negative feedback loop, ultimately resulting in a decrease in excitatory neurotransmitters, a parallel to the effects of alcohol [14].

While Baclofen monotherapy for AWS has not yet been proven effective [15], it is currently under investigation as a component of combination therapy. In a recent study [16] exploring the combination of baclofen and diazepam, researchers tested the efficacy of baclofen at 30 mg/day and 60 mg/day versus a placebo. The study measured the need for diazepam during withdrawal and found that 32.0% of patients on baclofen 60 mg/day required additional diazepam, compared to 72.0% on the placebo. Additionally, patients on baclofen needed significantly less diazepam compared to those on placebo. Adverse events were similar between the baclofen and placebo groups.

In another study comparing the length of stay of patients with AWS between the gabapentin/baclofen combination and lorazepam, the mean length of stay in the gabapentin/baclofen group was significantly shorter compared with the benzodiazepine group [17].

#### 3.2.2. Barbiturates

##### Phenobarbital

Barbiturates exert their effects by prolonging the opening of chloride channels, suppressing the central nervous system by binding to GABA-A receptor subunits, and maintaining a steady flow of chloride ions into neuronal cells [18]. Phenobarbital is the preferred barbiturate due to its short onset of action and long half-life. It can be used as monotherapy or in conjunction with benzodiazepines where benzodiazepines fail or refractory DTs are a concern [19].

A randomized controlled trial (RCT) involving 198 patients investigated the impact of a single intravenous dose of phenobarbital in conjunction with a lorazepam-based alcohol withdrawal protocol on intensive care unit (ICU) admissions for emergency department patients experiencing acute alcohol withdrawal. Those who received phenobarbital exhibited a lower rate of ICU admissions (8.0% vs. 25.0%) compared to the placebo group [20].

A meta-analysis of twelve studies and 1934 patients who presented to the ED with AWS analyzed the rate of intubation among patients who received phenobarbital compared with benzodiazepines as the primary outcome and rates of seizures, hospital, and ICU length of stay as secondary outcomes. The results did not differ between the benzodiazepine and phenobarbital groups [21].

A recent RCT comparing symptom-triggered benzodiazepine with phenobarbital in patients with severe acute AWS was completed in July 2023 and results are currently pending [22].

Evidence for the use of phenobarbital as monotherapy is limited and further studies are needed.

#### 3.2.3. Anesthetics

##### Ketamine

Ketamine, a dissociative anesthetic that functions as an NMDA antagonist, has been in clinical practice since the 1960s. Over the past decade, it has been investigated as a potential treatment for AUD [23]; however, studies investigating ketamine for AWS are limited. No RCTs were identified in the existing literature.

In a retrospective study involving 63 patients admitted to the ICU and diagnosed with DTs, those treated with symptom-triggered benzodiazepines, phenobarbital, plus IV ketamine had a significantly lower intubation rate and spent fewer days in the ICU [24].

In another retrospective study of 30 patients receiving ketamine adjunctively with a lorazepam infusion for severe alcohol withdrawal, significant decreases in lorazepam infusion rates were observed at 24 h after ketamine initiation. There were no documented central nervous system adverse effects [25].

##### Propofol

Propofol functions via GABA-A receptor agonism and targets a separate binding site from benzodiazepines. Additionally, it reduces glutamatergic activity by blocking NMDA receptors.

A comprehensive review [26] concluded that propofol for AWS yields mixed results, with propofol being comparable to benzodiazepine monotherapy for severe alcohol withdrawal. Dosages from 5.0 to 100.0 μg/kg/min have demonstrated effectiveness in reducing AWS symptoms, but its use is often associated with the frequent development of hypotension and the need for mechanical ventilation. Patients receiving propofol tend to experience longer durations of mechanical ventilation and hospital stay, possibly indicating more resistant cases of AWS. When compared to dexmedetomidine as adjuncts in AWS, both agents demonstrate similar benzodiazepine- and haloperidol-sparing effects. Dexmedetomidine is associated with more instances of bradycardia, while propofol is linked to more instances of hypotension. Propofol is considered in specific populations, particularly those already requiring mechanical ventilation or experiencing refractory symptoms.

In a recent retrospective study [27] on severe AWS in the ICU, the combination of propofol and dexmedetomidine demonstrated a higher reduction in CIWA-Ar scores within 24 h compared to their individual use. This combination was associated with a shorter hospital stay by approximately 3 days compared to propofol alone and 4 days compared to dexmedetomidine alone. The ICU stay was also reduced by about 1 day compared to dexmedetomidine alone and 1.5 days compared to propofol alone. However, these results did not reach statistical significance due to a small population size.

#### 3.2.4. Anticonvulsants

##### Gabapentin

Gabapentin, a structural analog of GABA, functions by binding to calcium channels. This binding action inhibits calcium influx, leading to a reduction in the release of excitatory neurotransmitters [28].

In a recent RCT involving 88 adults at risk of complicated AWS, participants were randomized to receive either a fixed-dose gabapentin taper or continued benzodiazepine administration based on CIWA-Ar. The primary outcome, length of stay, was shorter in the gabapentin group, though not statistically significant. Both groups received benzodiazepines before randomization, with the gabapentin group receiving less than half the amount compared to the benzodiazepine group. Secondary measures, including seizures, DTs, ICU transfer, and patient-reported symptoms, showed no statistical differences between the two groups [29].

A meta-analysis involving 2030 patients investigated the efficacy of gabapentin as a potential substitute for benzodiazepine use in treating acute alcohol withdrawal symptoms in hospitalized patients. The analysis found no significant differences in time to symptom resolution, the amount of benzodiazepines administered, withdrawal-related complications, or hospital length of stay between gabapentin-treated and benzodiazepine-treated groups. However, there was a notable difference in the rate of symptom resolution favoring gabapentin-treated patients. Subgroup analyses, particularly for severe AWS patients, indicated a significant decrease in hospital length of stay and a reduction in benzodiazepine administration in gabapentin-treated patients [30].

Gabapentin can also be used for ambulatory management of mild AWS. In an RCT involving 100 individuals seeking outpatient treatment for AWS, two doses of gabapentin were compared with lorazepam over a 4-day period. The study found that high-dose gabapentin was statistically superior but clinically similar to lorazepam in reducing alcohol withdrawal severity. During treatment, participants receiving lorazepam had a higher probability of drinking on the first day of dose decrease and the second day off medication compared to those receiving gabapentin. Post-treatment, gabapentin-treated participants had a lower probability of drinking during the follow-up period and also exhibited less craving, anxiety, and sedation compared to lorazepam-treated participants [31].

While gabapentin has been employed in the ambulatory management of AWS, the current literature does not support its use as a monotherapy for moderate to severe AWS. Nevertheless, gabapentin appears to be beneficial when used as an adjunct medication in inpatient settings.

##### Valproate

Studies from the 1980s have identified the potential merit of using valproate, particularly in ambulatory detox settings. However, these studies also concluded that the adverse effects associated with valproate use may outweigh the benefits [32,33].

A phase 4 clinical trial was conducted to assess valproic acid in preventing symptoms of AWS. The study compared benzodiazepine use in trauma patients who received benzodiazepines based on CIWA-Ar scores with patients who received prophylactic valproic acid therapy in addition to benzodiazepines. Although the trial concluded in 2020, the results have not yet been published [34].

#### 3.2.5. Alpha-2-Agonists

##### Dexmedotimidine and Clonidine

Dexmedotimidine is a newer agent which elicits sedation, anxiolysis, and sympatholysis by stimulating central presynaptic a2-autoreceptors. Importantly, since it does not activate GABA or opioid receptors, there is no respiratory compromise [35].

A systematic review investigated the effectiveness of dexmedetomidine as an adjunct to benzodiazepine-based therapy versus benzodiazepine-based therapy alone in reducing delirium severity associated with AWS in adult ICU patients. The analysis of four studies, comprising 55 patients, demonstrated that the addition of dexmedetomidine to benzodiazepine-based therapy significantly lowered CIWA-Ar scores, indicating a positive impact on delirium severity in AWS ICU patients [36].

Dexmedotimidine was also explored as an adjunct for the management of DTs in AWS. In the study, the use of dexmedetomidine as a second-line medication for severe alcohol withdrawal delirium, following local sedation protocol, prevented the need for anesthesia and intubation in all subjects [37].

In a recent small study involving 30 patients, where clonidine was administered alongside the standard alcohol withdrawal medication protocol, the clonidine group exhibited statistically significantly lower CIWA-Ar scores after the 5th day of treatment [38].

While the use of α2-agonists like clonidine and dexmedetomidine may be beneficial in lowering hypertension, tachycardia, and benzodiazepine requirements, they pose a minimal risk of respiratory depression. However, it is important to note that mechanistically, these agents lack antiepileptic properties and may not effectively prevent the onset of alcohol withdrawal delirium.

#### 3.2.6. Phosphodiesterase-4 Inhibitors

##### Ibudilast

Ibudilast is a phosphodiesterase inhibitor that suppresses proinflammatory cytokines. Excessive alcohol use has been shown to increase inflammation [39]. Therefore, it is hypothesized that ibudilast can be helpful in AUD due to its anti-inflammatory and pro-neutrophilic effects [40].

In an RCT involving 52 patients exploring the efficacy of ibudilast in the natural environment, with a focus on withdrawal-related dysphoria as a moderator, patients were randomly assigned to receive either ibudilast or a matched placebo. Patients reported their mood and craving levels through daily diary assessments over two weeks. While ibudilast did not significantly impact stimulation or sedation levels, it did moderate the influence of daily stimulation on drinking. Additionally, it reduced alcohol-induced cravings, particularly in individuals without withdrawal-related dysphoria, where it also tempered changes in the urge to drink and positive mood [41].

Studies investigating ibudilast for AWS are scarce in the literature. Nevertheless, this medication has displayed promising results, warranting further investigation.

#### 3.2.7. Antipsychotics

The use of antipsychotics, specifically haloperidol, is recommended only in combination with benzodiazepines, particularly in ICU and for patients experiencing DTs or those with comorbid psychotic disorders [42,43]. Regarding the use of antipsychotics for AWS, no recent clinical trials were identified in the literature. In the selection of medications, it should be remembered that antipsychotics are associated with QT prolongation and a decreased seizure threshold.

#### 3.2.8. Sodium Oxybate (SMO), Gamma-Hydroxybutyrate (GHB)

Sodium oxybate, also known as SMO or GHB, is approved for the treatment of AUD in Italy and Austria. GHB has a low binding affinity to GABA-B receptors, while it has a high binding affinity to receptors specifically designed for GHB. GABA-B receptors are believed to be the main drivers of the pharmacological effects of externally administered GHB. It affects the GABA system both directly by acting as a partial agonist for GABA-B receptors, and indirectly by leading to the production of GABA from GHB [44].

In a meta-analysis of 13 RCTs, a 50 mg dose of GHB was found to be more effective than a placebo in managing AWS. GHB was associated with mild side effects including transient vertigo. When compared to clomethiazole at the same dose, it was also more effective in reducing withdrawal symptoms [45].

A different study with 126 patients with AUD compared the effectiveness of GHB and oxazepam in treating uncomplicated AWS over 10 days. Both groups showed a significant reduction in AWS symptoms (CIWA-Ar scores), with no significant differences between the two treatments. Both GHB and oxazepam were well tolerated, and no severe side effects were reported [46].

There is a scarcity of data on GHB’s efficacy for AWS, and the recent literature does not present any new studies, underscoring the importance of conducting additional clinical studies.

Please refer to Table 1 for a summary of medications for AWS.

### 3.3. Treatment Modalities for Alcohol Use Disorder

Only three medications, disulfiram, acamprosate, and naltrexone are approved by the FDA for treatment of AUD. Additionally, The VA/DoD practice guidelines recommend the use of topiramate in AUD, while the APA guidelines suggest topiramate and gabapentin in addition to the three FDA-approved medications [9,47].

#### 3.3.1. FDA-Approved Medications

##### Naltrexone

Naltrexone treatment has a longstanding history [48], but its effectiveness has been improved with the introduction of a long-acting injectable form. This form of Naltrexone is used for treating AUD, with the goal of improving patient compliance and overall treatment outcomes [49]. It exerts its effects through mu-opioid receptor antagonism, reducing cravings, the reinforcing effects of alcohol, and binge drinking [50].

In a meta-analysis of 53 studies, it was found that the number needed to treat with naltrexone to prevent return to heavy drinking was 12. Patients who received medical management along with naltrexone, a combined behavioral intervention, or both, showed improved drinking outcomes compared to those who received a placebo. Additionally, oral naltrexone at a dosage of 50 mg/day was associated with a reduced likelihood of returning to drinking [51].

The most common side effects of naltrexone include nausea, dizziness, constipation, headache, and fatigue. Additionally, it is contraindicated in patients with active opioid use, acute hepatitis, and liver failure.

##### Disulfiram

Disulfiram acts by inhibiting aldehyde dehydrogenase. If alcohol is consumed, this leads to the accumulation of acetaldehyde. This results in an unpleasant reaction which includes sweating, flushing, nausea, and tachycardia.

In a meta-analysis of 22 studies, disulfiram demonstrated a higher success rate in promoting abstinence compared to placebo in open-label trials [52]. There is a debate about whether disulfiram should be employed as a primary treatment for AUD or if its use should be reserved as an adjunct for maintaining abstinence [53].

The side effects of disulfiram encompass hepatotoxicity, fatigue, drowsiness, and headache. Its utilization is contraindicated in individuals with coronary artery disease and psychosis.

##### Acamprosate

Acamprosate acts as an NMDA receptor antagonist and modulates mGluR5 receptors [54].

A large-scale meta-analysis, comprising 27 RCTs, found that acamprosate was effective in prolonging abstinence but did not reduce rates of binge drinking [51].

Another meta-analysis, based on 24 RCTs involving 6915 participants, revealed that acamprosate, when compared to a placebo, significantly reduced the risk of any drinking and increased cumulative abstinence. Secondary outcomes, such as gamma-glutamyltransferase and heavy drinking, did not reach statistical significance [55].

Acamprosate may cause side effects such as anxiety, diarrhea, fatigue, and headache. Importantly, it is contraindicated in individuals with severe renal dysfunction.

Please refer to Table 2 for summary of FDA-approved AUD medications.

#### 3.3.2. Non-FDA Approved Treatment Modalities

##### Baclofen

Research on the use of baclofen for treating AUD has yielded conflicting results. In a 16-week RCT with 120 participants diagnosed with AUD, the effects of 30 and 90 mg/day of baclofen were compared to a placebo. The study examined the influence of dose, gender, and pretreatment drinking levels. The key findings indicated a significant impact of baclofen, particularly at the 90 mg/day dose, leading to a reduction in the percentage of heavy drinking days and an increase in abstinent days. Notably, higher doses were less well tolerated in female participants [56].

In a meta-analysis comparing the effectiveness of baclofen to placebo and acamprosate on relapse rates, frequency of alcohol use, and frequency of heavy drinking days, there were no significant differences between baclofen and placebo. Similarly, the number of participants experiencing at least one adverse event, the dropout rate and dropout due to adverse events did not differ significantly. Overall, the review suggests that the use of baclofen as a first-line treatment for AUDs remains uncertain due to the lack of conclusive evidence and heterogeneity among studies [57].

##### ASP8062

ASP8062 is a novel agent which acts as a positive allosteric modulator of GABA-B, similar to baclofen, which is an orthosteric GABA-B receptor agonist [58]. Currently, despite some promising results, more clinical trials are needed to further investigate the potential of this agent in the treatment of AUD.

In a recent animal study comparing ASP8062 and baclofen in male and female rats to evaluate their effects on alcohol self-administration, both compounds demonstrated a reduction in alcohol consumption in both genders. Notably, ASP8062 was more effective in males, while baclofen had a greater impact on females. ASP8062 did not affect locomotor activity, whereas baclofen decreased activity in males (at 3.0 mg/kg) but increased it in females (at 1.0 mg/kg) [59].

In two phase 1 human clinical trials investigating safety and tolerability, ASP8062 demonstrated good tolerability, with no observed drug-related effects on safety, cognitive function, withdrawal symptoms, or suicidal ideation [60].

##### Sodium Oxybate (SMO), Gamma-Hydroxybutyrate (GHB)

In an RCT involving 509 patients with AUD, a new formulation of GHB was assessed for abstinence maintenance. The primary endpoint and the percentage of days abstinent did not differ between groups. However, significant improvements were observed in several secondary measures. Post-hoc analysis highlighted a notable benefit in severe AUD patients, showing increased abstinence. The study reported no safety concerns [61].

In a meta-analysis of 13 RCTs, GHB demonstrated benefits in promoting abstinence, controlled drinking, and reducing relapses and daily alcohol consumption. It surpassed naltrexone and disulfiram in terms of abstinence and combining GHB with naltrexone yielded even better results. Moreover, GHB was more effective in reducing alcohol craving compared to a placebo and disulfiram [45].

GHB has shown promising results in the treatment of AUD in various clinical studies. However, this agent is not approved by the FDA. While there are previous studies, the current literature lacks recent clinical studies, highlighting the need for further research.

##### Topiramate

Topiramate inhibits voltage-dependent sodium channels, enhances the inhibitory activity of GABA, and is believed to decrease dopaminergic activity in reward circuits in the brain, ultimately reducing cravings [62]. Topiramate is recommended by the VA/DoD and APA practice guidelines for the treatment of AUD.

In a 14-week RCT involving 371 individuals with AUD, patients were administered either up to 300 mg/d of topiramate or a placebo, alongside weekly compliance enhancement intervention. Topiramate exhibited greater efficacy than placebo in reducing the percentage of heavy drinking days, with a mean difference of 8.44%. However, compared to placebo, topiramate was associated with higher rates of adverse events, including paresthesia, taste perversion, anorexia, and difficulty with concentration [63].

In a different study comparing topiramate with baclofen, following a 1-week detox, 94 patients were randomly assigned to either baclofen or topiramate for a 1-month follow-up. Baclofen exhibited a significant improvement in obsessive and compulsive drinking scale scores compared to topiramate, with 61.22% achieving complete abstinence versus 37.78% in the topiramate group. Baclofen was better tolerated, as indicated by a lower dropout rate (24.49%) compared to topiramate (33.33%) [64].

In a recent meta-analysis reviewing 13 RCTs comparing topiramate to a placebo for treating AUD, topiramate demonstrated effectiveness in reducing heavy drinking days and weeks, decreasing alcohol craving, prolonging abstinence, and lowering gamma-glutamyl transferase levels. The analysis also suggested potential benefits in reducing anxiety. However, its efficacy in addressing AWS, preventing relapse, and reducing depressive symptoms remained inconclusive. Notably, topiramate was associated with a significantly higher prevalence of paresthesia, drowsiness, and memory impairment compared to the placebo [65].

##### Gabapentin

In an RCT comparing gabapentin to a placebo in 90 individuals with AUD and recent alcohol withdrawal symptoms, participants received gabapentin (up to 1200 mg/d) or a placebo along with medical management visits over a 16-week period. The gabapentin group achieved more non-heavy drinking days (27.0% vs. 9.0%) and total abstinence (18.0% vs. 4.0%) compared to the placebo group. These effects were more pronounced in participants with a history of high alcohol withdrawal symptoms. Although gabapentin caused more dizziness, it did not impact its efficacy. The study suggests that gabapentin may be more effective, especially in individuals with a history of high alcohol withdrawal symptoms [66].

In a different study involving 346 individuals with AUD, participants were randomly assigned to receive either extended-release gabapentin (GE-XR) at 600 mg twice a day or a placebo, along with a computerized behavioral intervention over a 6-month period. The primary outcome measure, the percentage of subjects with no heavy drinking days, did not significantly differ between the GE-XR and placebo groups. Additionally, no clinical benefits were observed for various drinking measures, alcohol craving, alcohol-related consequences, sleep problems, smoking, and depression/anxiety symptoms [67].

In a meta-analysis of seven RCTs assessing outcome measures such as complete abstinence, relapse to heavy drinking, percent days abstinent, percent heavy drinking days, drinks per day, and gamma-glutamyl transferase concentration, gabapentin showed statistically significant efficacy only in reducing the frequency of heavy drinking, with inconclusive results for other outcomes [68].

Overall, gabapentin has demonstrated efficacy in reducing heavy drinking behavior. However, it is crucial to consider potential side effects and the risk of misuse, particularly in patients with opioid use disorder [69].

##### Ondansetron

Ondansetron is an antiemetic medication with 5-HT3 receptor blocking effects. Various clinical studies have shown promising results.

In a double-blind, placebo-controlled study, ondansetron and sertraline were compared for their anti-craving effects. The study revealed that a genetic variation in the serotonin 5-HT3 transporter (5-HTTLRP) influenced the effectiveness of ondansetron therapy, demonstrating a direct link between genetic polymorphism and the response to pharmacological treatment [70].

In a different RCT involving 107 patients, individuals received either naltrexone, ondansetron, a combination of both, or placebo for a week. When exposed to alcohol-related cues, the combination of naltrexone and ondansetron effectively reduced alcohol craving. Furthermore, both medications, whether administered alone or in combination, decreased activation of the ventral striatum in response to alcohol cues [71].

In a different study evaluating the effectiveness and safety of a 16 mg/day dosage of ondansetron in outpatient settings, ondansetron proved superior to placebo, showcasing a reduction in the proportion of heavy drinking days (7.8% vs. 11.7%) [72].

Based on the current literature, ondansetron can be beneficial in decreasing heavy drinking; however, it is important to note potential side effects, including fatigue, constipation, diarrhea, and QT prolongation, which should be carefully considered.

##### Psychedelics

Classical psychedelics are a category of substances that includes lysergic acid diethylamide (LSD), psilocybin, ayahuasca (DMT), and mescaline. These compounds induce altered states of consciousness primarily by interacting with the 5-HT2A receptors [73].

Non-classic psychedelics, on the other hand, work through various mechanisms. For example, ketamine blocks NMDA receptors, MDMA influences serotonin and dopamine, ibogaine has multiple targets, salvinorin A stimulates kappa-opioid receptors, and delta-9-tetrahydrocannabinol (THC), found in cannabis, acts on cannabinoid receptors. These substances, like classic psychedelics, can change consciousness significantly, but they have distinct mechanisms and effects [73].

In the mid-20th century, clinicians frequently used psychedelics to treat conditions like schizophrenia, anxiety, mood disorders, and addiction. LSD, in particular, was popular for addressing AUD. These substances were found to reduce alcohol cravings and consumption. Scientists were exploring whether its ability to enhance self-awareness could be beneficial in therapy for individuals with a history of alcohol misuse [74]. However, in 1965, LSD was banned in the US, and Sandoz also ceased providing psychedelic drugs for research. Similar restrictions were placed on other psychedelics as well. Recently, there has been a resurgence of interest in their therapeutic potential.

##### LSD

The most recent RCTs evaluating LSD for AUD date back to the 1960s and 1970s. A recent meta-analysis, which examined six RCTs, revealed that a single LSD dose had a significant and positive impact on reducing alcohol misuse in the first 1 to 12 months following treatment. However, this effect did not remain statistically significant beyond the 12-month mark [75].

Animal studies on LSD are also very limited. In a 2018 study, researchers explored the influence of LSD on alcohol consumption in mice. They administered two different LSD doses (25 and 50 μg/kg) and observed that the group treated with 50 μg/kg of LSD significantly reduced both alcohol intake and preference compared to the control group. This reduction was sustained for 46 days after LSD administration, suggesting a biologically mediated effect beyond psychological factors [76]. However, in a recent study with rats aimed at investigating the effects of psilocybin/LSD on the alcohol deprivation effect, neither of the two LSD doses used (0.08 and 0.32 mg/kg) had any impact on alcohol relapse in animal models [77].

##### Psilocybin

A recent animal study investigated the impact of various psilocybin/LSD treatment schedules on relapse-like drinking in rats. High doses and chronic microdosing of psychedelics showed no lasting effects on relapse behavior [77].

In a recent clinical trial, the effectiveness of combining psychotherapy with psilocybin was compared to an active placebo for treating AUD. Participants received medication in two sessions at weeks 4 and 8, along with 12 weeks of therapy. Over the 32-week double-blind follow-up period, starting from the first dose, the psilocybin group experienced a twofold reduction in heavy drinking compared to the diphenhydramine group (number needed to treat: 4.5). The percentage of days with heavy drinking in the psilocybin-treated group was only 41% of that observed in the diphenhydramine-treated group. Furthermore, the psilocybin group had three times higher chances of having no heavy drinking days and fewer alcohol-related adverse consequences compared to the diphenhydramine group [78].

### 3.4. 4,5-Trimethoxyphenethylamine (Mescaline)

Mescaline, a natural alkaloid of the phenyl-alkylamine class, is among the oldest psychedelics known, with a history dating back 5700 years. Historically, it has been used in religious rituals [79].

In one of the earliest studies published in 1974, mescaline was investigated among Indigenous Peoples/American Indians with AUD through a therapeutic approach involving group meetings, cultural therapy, and participation in church meetings, some involving peyote/mescaline. Participants, consuming an average of 500 mg mescaline during church ceremonies, discussed alcoholism and expressed emotions, leading to cathartic experiences that aided in overcoming AUD. The study claimed that, for many American Indians, a single peyote meeting served as a pivotal turning point [80].

A 2021 international survey involving 452 participants explored the impact of mescaline use in non-clinical settings on mental health. Self-reports revealed significant improvements in depression (86.0%), anxiety (80.0%), post-traumatic stress disorder (76.0%) and AUD (76.0%). The findings of this study suggest a potential positive association between mescaline use and the alleviation of various mental health symptoms [81].

#### Ketamine

Multiple combinations of ketamine, psychotherapy, and other psychedelics for the treatment of AUD have been investigated since the early 90s [82,83].

In a recent study, 40 participants were randomly assigned to either ketamine or the active control midazolam for intravenous administration during the second week of a 5-week outpatient motivational enhancement therapy regimen. Ketamine significantly increased the likelihood of abstinence, delayed time to relapse, and reduced the likelihood of heavy drinking days compared to midazolam. The ketamine infusions were well tolerated, with no participants removed from the study due to adverse events [84].

In a different study with 96 patients with AUD, participants were assigned to receive either ketamine infusions with psychotherapy, saline infusions with psychotherapy, ketamine infusions with alcohol education, or saline infusions with alcohol education. At the 6-month follow-up, the ketamine group, especially those with additional therapy, showed a significantly higher number of days abstinent compared to the placebo group. However, there was no significant difference in relapse rates between the ketamine and placebo groups, and the treatment was well tolerated [85].

### 3.5. Phosphodiesterase-4 Inhibitors

#### 3.5.1. Ibudilast, Apremilast

Ibudilast for AUD has demonstrated promising results in several early studies and was well tolerated. Nevertheless, further clinical trials are required to better understand its potential as a standalone treatment or as part of a combination therapy.

A study involved a double-blind, placebo-controlled laboratory investigation of ibudilast (50 mg BID) in individuals with mild-to-severe AUD. Participants (*n* = 24) underwent two 7-day outpatient protocols, receiving either the medication or a placebo. While ibudilast was well tolerated, it did not significantly affect subjective responses to alcohol. However, particularly among those with higher depressive symptoms, there were decreased cravings and mitigated stimulant and mood-altering effects of alcohol compared to the placebo [86].

In an RCT, participants with AUD were given ibudilast or a placebo for two weeks. The ibudilast group exhibited lower inflammation markers in their brains and bodies. Interestingly, a link was observed between C-reactive protein and choline compound (Cho) levels, and Cho levels in the brain predicted drinking behavior in the following week [87].

In another RCT involving apremilast, the research encompassed both animal and human trials, demonstrating that apremilast effectively reduced excessive alcohol consumption across various models and in individuals with AUD. The nucleus accumbens (NAc) region was identified as critical for regulating alcohol-related behaviors, and apremilast was found to impact the functional activity of specific neural cell types within the Nac. Additionally, apremilast exhibited a significant reduction in drinking, surpassing the efficacy of existing FDA-approved treatments for AUD, such as acamprosate and naltrexone [88].

#### 3.5.2. Ghrelin; PF-5190457

Ghrelin is primarily recognized for its function in regulating appetite through the hypothalamic arcuate nucleus [89]. Furthermore, it is being studied for its potential involvement in reward-seeking and stress-related behaviors. Its interaction with neurobiological circuits, such as the cholinergic–dopaminergic pathway, hints at its possible role in drug-seeking and addictive behaviors [90]. PF-5190457, an inverse agonist for the ghrelin receptor discovered by Pfizer in the mid-2010s as a clinical candidate for diabetes, effectively suppresses GHS-R1a activity, both at its baseline function and in response to ghrelin [91]. Additionally, ghrelin has anti-inflammatory effects and has been shown to inhibit the release of pro-inflammatory cytokines [92].

In a small initial clinical trial involving 12 heavy drinkers, PF-5190457, when compared to a placebo, lessened alcohol cravings and reactivity to alcohol-related cues. Furthermore, when used in conjunction with alcohol, PF-5190457 proved safe and well tolerated, showing no adverse interactions between the drug and alcohol [93].

Despite promising results from various animal and clinical studies, no recently conducted clinical trials were identified in the literature. Additional studies are required to assess the potential use of this agent for treating AUD.

#### 3.5.3. GLP-1 Receptor Agonists

Reward mechanisms, important in both AUD and food overconsumption behavior, are driven by a disparity between expected effects and reduced rewards. This is believed to be associated with lower dopamine receptor levels in the striatum, impacting frontal brain regions and causing imbalances in motivation, inhibition, and stress reactivity [94].

In the intestine and pancreas, GLP-1 is produced and secreted upon meal ingestion. Receptors for GLP-1, which mediate the physiological and behavioral effects of GLP-1, have been discovered in regions of the brain linked to reward and addiction, including the ventral tegmental area (VTA) and the NAc [95].

After a meal is consumed, there is a swift increase in GLP-1 levels and the enzymes DPP-IV and neutral endopeptidase break down GLP-1. Consequently, some of GLP-1 manages to reach the brain once it is released from the intestine [96].

In a study comprising four human genetic association studies, researchers found a nominal association between the 168Ser allele (rs6923761) and AUD. The 168 Ser/Ser genotype was linked to increased alcohol administration and higher breath alcohol measures in a human laboratory experiment. Furthermore, this genotype exhibited an elevated blood-oxygen-level dependent response in the right globus pallidus when participants were notified of a high monetary reward [97].

A recent study compared GLP-1 receptor (GLP-1R) expression in post-mortem brain tissues between individuals with AUD and controls. Individuals with AUD exhibited a significantly higher expression of GLP-1R in the hippocampus. Exploratory analyses revealed correlations between GLP-1R gene expression levels and behavioral measures of alcohol consumption [98].

In an animal study, the impact of nine or five weeks of weekly dulaglutide administration on ethanol intake in male and female rats was investigated. Both durations resulted in reduced ethanol intake and preference. After treatment discontinuation, the decrease in ethanol consumption persisted in males but not females [99].

Another animal study explored the impact of the GLP-1 receptor agonist Exendin-4 (Ex4) on alcohol-induced reward, intake, and seeking behavior in rodents. Ex4 mitigated alcohol-induced effects in mice, including locomotor stimulation and dopamine release. In mice, both acute and chronic Ex4 treatment eliminated conditioned place preference for alcohol. Additionally, Ex4 decreased alcohol-seeking behavior [100].

In a different animal study, researchers investigated the impact of semaglutide, a GLP-1 analogue on alcohol consumption in mice and rats. The results showed that semaglutide significantly reduced binge-like alcohol drinking in both male and female rats, and the effect was dose-dependent. The study also explored semaglutide’s influence on dependence-induced alcohol intake in rats. Importantly, the effects were not specific to alcohol, as semaglutide also reduced the intake of various non-alcoholic solutions, suggesting a broader role in suppressing behaviors related to appetite, thirst, and palatability. The research suggested that semaglutide influenced GABAergic synaptic transmission in brain areas associated with alcohol-related behaviors [101].

In a case series, six patients treated with semaglutide for weight loss who had positive screenings for AUD on the Alcohol Use Disorder Identification Test (AUDIT) before starting semaglutide therapy were analyzed. The results showed that all six patients experienced a significant reduction in AUD symptomatology, as evidenced by an improvement in AUDIT scores (mean decrease of 9.5 points, *p* < 0.001) following semaglutide therapy [102].

In a nationwide cohort study in Denmark spanning from 2009 to 2018, researchers investigated the association between GLP-1 agonists and the risk of alcohol-related events, including hospital contacts with a main diagnosis of alcohol use disorders, receiving registered treatments for AUD, or purchase of the benzodiazepine chlordiazepoxide. The study included 38,454 new users of GLP-1 agonists and 49,222 users of dipeptidyl peptidase 4 inhibitors (DPP4). The findings revealed that initiating GLP-1 treatment was linked to a significantly lower risk of alcohol-related events compared to initiating DPP4 treatment during the first three months of follow-up [103].

In a recent RCT involving 127 participants, exenatide did not significantly reduce heavy drinking days compared to a placebo. However, it did decrease fMRI alcohol cue reactivity in brain regions associated with addiction and lowered dopamine transporter availability. Notably, a subgroup of obese patients with a BMI over 30 kg/m^2^ showed reduced heavy drinking and total alcohol intake, with primarily gastrointestinal side effects [104].

Overall, GLP-1 agonists have demonstrated very promising results in the treatment of AUD. Additional clinical trials exploring the effects of newer GLP-1 analogous in AUD are needed. With ongoing research, there is a strong likelihood that this medication group will become integrated into the standard treatment for AUD.

#### 3.5.4. Noninvasive Neural-Circuit-Based Interventions

##### Transcranial Magnetic Stimulation (TMS)

It has been demonstrated that drug-related behaviors extend beyond the dopamine-rich striatum. Cortical regions linked to the striatum, such as the dorsolateral prefrontal cortex (PFC), dorsal cingulate cortex, and posterior parietal cortex, play a crucial role in controlling actions and decisions. Furthermore, the ventral PFC network, encompassing the medial PFC, orbitofrontal cortex, insular cortex, and ventral anterior cingulate cortex, influences emotional processing and limbic arousal in addiction [105].

In the early 2000s, two influential studies demonstrated a causal relationship between the application of transcranial magnetic stimulation (TMS) on the prefrontal cortex and dopamine binding in the caudate nucleus, leading to an increase in extracellular dopamine and glutamate in the ventral striatum by TMS [106,107]. An alternative method involves reducing activity in areas linked to alcohol cue reactivity, such as the medial prefrontal cortex (MPFC), striatum, insula, and anterior cingulate cortex (ACC) [108].

In a double-blind trial, 51 patients with AUD received either active or sham deep transcranial magnetic stimulation (dTMS) treatment targeting midline frontocortical areas. The active dTMS group had reduced craving and fewer heavy drinking days during follow-up, suggesting the potential effectiveness of dTMS in treating AUD, with associated changes in brain connectivity [109].

A different recent clinical trial investigated the use of continuous theta burst stimulation (TBS) to reduce drinking behavior and brain reactivity to alcohol cues in individuals with AUD. Real TBS sessions increased the likelihood of remaining enrolled in the study and achieving sobriety after 3 months. Additionally, real TBS resulted in reduced brain reactivity to alcohol cues, including decreased connectivity between the medial prefrontal cortex (MPFC) and the striatum and insula [110].

In two single-blinded active sham-controlled experiments involving 49 participants with AUD and cocaine use disorder, researchers used continuous theta burst stimulation (cTBS) to assess neural reactivity to drug/alcohol cues compared to neutral cues. The study revealed a significant interaction between treatment (real/sham) and time (pre/post), indicating that cue-related functional connectivity was significantly attenuated following real cTBS versus sham cTBS. This effect was observed across various regions, including the ventral striatum, caudate, putamen, insula, and anterior cingulate cortex [108].

##### Deep Brain Stimulation (DBS)

Animal models have shown that both ethanol intake and anticipation prompt dopamine release in the NAc, impacting the mesocorticolimbic system associated with craving, reward, and behavioral control [111]. The NAc is a crucial region in AUD models [112]. Neuroimaging studies consistently demonstrate that exposure to alcohol cues increases activation in the NAc and striatum, which is correlated with subjective alcohol craving [113].

In a phase 1 trial, six patients with severe, treatment-resistant AUD underwent NAc deep brain stimulation (NAc-DBS) therapy, resulting in a reduction in cravings and alcohol-related issues. Clinical improvement was associated with decreased NAc metabolism, disrupted connectivity to the visual cortex, and reduced dorsal striatum activation in response to alcohol cues [114].

In a recent RCT with 12 AUD inpatients, comparing DBS to sham stimulation over 6 months, the primary intention-to-treat analysis did not yield statistically significant results for continuous abstinence. However, secondary outcome analyses showed a significantly higher proportion of abstinent days, lower alcohol craving, and anhedonia in the DBS group. Furthermore, findings suggested that patients with high baseline alcohol craving, depression, and anhedonia responded positively to DBS [115].

##### Psychosocial Treatments

Psychosocial interventions, such as motivational psychotherapy, brief interventions, cognitive behavioral therapy (CBT), contingency management (CM), third-wave therapies (acceptance and commitment therapy and mindfulness-based approaches), residential programs, and Alcoholics Anonymous/12-Step Facilitation (AA/TSF) can be utilized in the treatment of AUD. The VA/DoD clinical practice guidelines recommend psychosocial treatments, including behavioral couples therapy, cognitive behavioral therapy, community reinforcement, motivational enhancement therapy, and 12-step facilitation, for patients with AUD [47].

##### Brief Interventions

Brief interventions are typically used in conjunction with screenings for unhealthy drinking. They involve a combination of motivational interviewing, feedback on drinking behavior, and coping strategies to help individuals change their drinking patterns. These interventions typically take 5 to 20 min and are recommended for all patients with risky alcohol use [116].

A systematic review, including 23 clinical trials, investigated behavioral counseling interventions for alcohol misuse in primary care. Brief multicontact interventions (10–15 min) showed effectiveness, reducing alcohol consumption by 3.6 drinks per week, with 12.0% fewer heavy drinking episodes and 11.0% more adhering to recommended limits over 12 months compared to controls [117].

Motivational interviewing (MI) is a type of brief intervention that utilizes open-ended questions, affirmations, reflective listening, and summarization as essential tools in its methodology.

A Cochrane systematic review analyzing 93 randomized controlled trials, involving 22,776 participants, revealed that MI led to a reduction in substance use post-intervention. MI demonstrated a standardized mean difference (SMD) of 0.48 compared to no intervention, indicating a small to moderate effect, while also showing potential improvements in treatment retention, with an SMD of 0.26 [118].

In an MI study conducted in Kenya involving 300 adults with problematic alcohol use, participants received immediate mobile MI (*n* = 89), in-person MI (*n* = 65), or delayed mobile MI (*n* = 76) for waiting-list controls. AUDIT scores were assessed, with the primary outcome being the difference in alcohol score between waiting-list controls and mobile MI 1 month after intervention. Results showed a significant improvement with mobile MI, with a difference of 2.88 points compared to waiting-list controls. Secondary outcomes indicated no significant difference between in-person and mobile MI at 1 month, while results at 6 months were inconclusive [119].

##### Cognitive Behavioral Therapy (CBT)

CBT is a structured, multi-session intervention aimed at addressing cognitive, emotional, and environmental factors contributing to substance use. It offers coping skills training to support individuals in attaining and sustaining abstinence or minimizing harm.

A meta-analysis of 30 randomized controlled trials evaluated the efficacy of CBT for alcohol or substance use disorders across different treatment approaches. Compared to minimal treatment (waitlist, brief psychoeducation), CBT demonstrated a moderate and significant effect size consistently across outcome types and follow-up periods. Contrast with non-specific therapy (supportive therapy, drug counseling) showed significant effects on consumption frequency and quantity at early follow-up, but not at late follow-up. However, CBT effects compared to specific therapy (motivational interviewing, contingency management) were consistently non-significant across outcomes and follow-up times [120].

There are currently no recent studies in the literature specifically investigating effects of CBT in AUD.

##### Contingency Management (CM)

CM involves techniques that are designed to promote abstinence and discourage drinking by offering small incentives.

In a study involving 82 non-treatment-seeking heavy drinkers, three phases, including observation, contingency management, and follow-up, were conducted over a 28-week period. The contingency management phase, lasting 12 weeks, involved participants being paid 50 Dollars weekly for not exceeding low levels of alcohol consumption. Transdermal alcohol monitors verified meeting contingency requirements, while other analyses relied on self-reported alcohol use. Results showed a significant decrease in self-reported drinking days and heavy drinking days during the contingency management phase. Furthermore, these reductions persisted or became more pronounced during the follow up phase [121].

In a different study, with 30 non-treatment-seeking heavy drinkers the contingency management group showed a higher percentage of days without detected drinking, with a longer consecutive period of abstinence (8.0 days vs. 2.9 days). The intervention group adhered to low-risk drinking guidelines four times more than the control group (31.1% vs. 7.1%) [122].

##### Alcoholics Anonymous/12-Step Facilitation (AA/TSF)

AA, a worldwide mutual support group, extends its reach to 181 countries and comprises millions of members. The original AA intervention is thought to be effective due to its emphasis on social fellowship and a 12-step program. These social components provide peer support, role modeling for successful recovery, close mentoring through sponsorship, and help reduce negative emotions such as shame and guilt [123].

A Cochrane meta-analysis of 27 studies, involving 10,565 participants, assessed the effectiveness of AA/TSF compared to psychological interventions like CBT for alcohol or substance use disorders. Findings demonstrated that manualized AA/TSF significantly improved continuous abstinence rates at 12 months compared to CBT. Non-manualized AA/TSF also showed similar performance to other interventions in terms of abstinence rates and drinking intensity at various follow-up periods [124].

##### Third-Wave Therapies

In third-wave therapies, acceptance and commitment therapy and mindfulness-based approaches, distress is not considered a direct result of cognition. Hence, these approaches avoid trying to control or alter thought content. These therapies argue that attempting to control unwanted thoughts can worsen distress and lead to maladaptive behaviors [125].

In a study with 262 military personnel, a one-week group-based acceptance and commitment therapy intervention was investigated compared to a waitlist control group. The intervention group exhibited improvements, including reduced alcohol consumption, anxiety, and stress [126].

In a different RCT, the isolated effects of 11 min of supervised mindfulness instruction were compared against a control relaxation in 68 patients with alcohol use. The mindfulness group exhibited a significant reduction in past-week alcohol consumption at the 7-day follow-up, while the relaxation group did not show a significant change [127].

Please see Table 3 for a summary of non-FDA approved treatment modalities for AUD.

## 4. Conclusions

Although the current gold standard treatment for AWS is benzodiazepines, emerging evidence indicates that a variety of pharmacological agents with diverse mechanisms of action could potentially substitute for or complement benzodiazepines in managing AWS. Current trends indicate that the use of phenobarbital in ICU settings and gabapentin in outpatient settings appears promising. With a deeper grasp of the pathophysiology and neurobiology of AWS, identifying alternative treatment options could be possible.

AUD poses a widespread public health concern, yet current treatment strategies prove insufficient, falling short of addressing the complexity of the issue. The need for more effective treatment approaches is urgent, but of equal importance is the fact that we have very few options for the prevention of the development of this condition. The ongoing discourse surrounding the integration of the concept of ‘preaddiction’ [128] offers a potential paradigm shift, suggesting a potential alternative to the mild or moderate substance use disorder criteria in DSM-5-TR. This concept proposes a more understandable label for a vulnerable phase during which preventive measures could be vital to mitigate the risk of severe consequences related to drug use before progressing to severe substance use disorder.

While commendable progress has been made in understanding AUD and developing novel and repurposed agents, along with other treatment modalities for this condition, the challenge remains unsolved. To date, no treatment modality has demonstrated a permanent cure for AUD. Exploring medications targeting different mechanisms, as well as combination approaches like integrating psychotherapy and psychedelics, appears promising. Further research should focus on conducting clinical and safety trials for promising treatment methods. Simultaneously, there is a pressing need to explore novel treatment strategies.

## Figures and Tables

**Table 1:** Medications for AWS.

Treatment Modality	Mechanism of Action	Results
GABA-A receptor agonists (Benzodiazepines)	Stimulation of GABA-A receptors	Gold standard for AWS; ↓ withdrawal severity; ↓ DTs
GABA-B receptor agonists (Baclofen)	↓ Excitatory neurotransmitter release; stimulation of GABA-B receptors	Effective as a benzodiazepine sparing agent; ↓ length of hospital stay
Barbiturates (Phenobarbital)	↑ duration chloride ion channel opening; ↓ glutamate signaling	No sufficient evidence for monotherapy; ↓ ICU admission and intubation rates when used with benzodiazepines
Anesthetics (Ketamine, propofol)	NMDA antagonism; GABA-A receptor agonism	No sufficient evidence for monotherapy; ↓ Intubation rates when used with benzodiazepines; useful in refractory DTs
Gabapentin	↓ GABA receptor mediated inhibitory post synaptic currents (IPSCS); voltage gated calcium channel blockage	No sufficient evidence for monotherapy; As an adjunctive in inpatient settings and outpatient management of AWS
GHB	GABA-B partial agonist	No recent studies; Some effectiveness in uncomplicated AWS
Alpha-2-agonists	↑ central presynaptic a2-autoreceptor stimulation; ↓ autonomic hyperactivity	Could be useful as adjunctive medications; ↓ delirium severity and intubation in ICU settings
Phosphodiesterase-4 inhibitors	↓ proinflammatory cytokines; Selective phosphodiesterase inhibition	No sufficient evidence; ↓ alcohol craving; positive mood effects
Antipsychotics	Dopamine antagonism	No recent studies; Useful in uncontrolled agitation, hallucinations

**Table 2:** FDA-approved AUD medications.

Treatment Modality	Mechanism of Action	Results
Naltrexone	Mu-opioid receptor antagonism	↓ cravings, reinforcing effects of alcohol, binge drinking; ↓ relapse
Disulfiram	ALDH inhibition resulting in acetaldehyde accumulation	↑ abstinence; questionable efficacy as monotherapy
Acamprosate	NMDA receptor antagonist; mGluR5 receptor modulation	↑ abstinence; not effective in heavy drinking

**Table 3:** Non-FDA approved treatment modalities for AUD.

Treatment Modality	Mechanism of Action	Results
Baclofen	↓ Excitatory neurotransmitter release; stimulation of GABA-B receptors	Conflicting clinical results ↓ heavy drinking days ↑ abstinent days
Asp8062	Allosteric modulator of GABA-B	↓ alcohol consumption in animal studies Good safety and tolerability in human studies
GHB	Partial agonist for GABA-B receptors; ↑ production of GABA from GHB	↑ abstinent days ↓ daily alcohol consumption ↓ withdrawal symptoms
Topiramate	Inhibits voltage-dependent sodium channels; ↑ inhibitory activity of GAB	↓ percentage of heavy drinking days ↑ increased percent days abstinent ↓ alcohol craving
Gabapentin	↓ GABA receptor mediated inhibitory post synaptic currents (IPSCS); blocks voltage gated calcium channels	Conflicting results ↑ non-heavy drinking days ↑ abstinent days Failed effectiveness in extended-release gabapentin (GE-XR)
Ondansetron	5-HT3 receptor blockage	↓ alcohol craving (in combination with naltrexone) ↓ percentage of heavy drinking days
LSD	Interaction with the 5-HT2A receptors	Old clinical trials from 70s Conflicting animal studies; ↓ alcohol intake and preference in rats
Psilocybin	Interaction with the 5-HT2A receptors	Psychotherapy, psilocybin combination: ↓ percentage of heavy drinking days Failed recent psilocybin/LSD microdosing in rats
Mescaline	Interaction with the 5-HT2A receptors	No RCTs Self-reported cathartic experiences leading to AUD alleviation ↓ self-reported daily alcohol consumption
Ketamine	NMDA antagonism	Psychotherapy, ketamine combination; ↓ percentage of heavy drinking days ↑ increased percent days abstinent No difference in relapse rates
Ibudilast; apremilast	↓ proinflammatory cytokines; Selective phosphodiesterase inhibition	↓ alcohol craving ↓ neural cue-reactivity ↓ daily alcohol consumption
Pf-5190457	Ghrelin receptor inverse agonism	↓ alcohol craving ↓ neural cue-reactivity
Dulaglutide, Exendin-4 (Ex4), exenatide, Semaglutide	GLP-1 receptor agonism	↓ alcohol intake and preference, locomotor stimulation and dopamine release in rats ↓ alcohol cue reactivity, heavy drinking and total alcohol intake in obese patients
TMS, DBS	Electrical current induction to depolarize neurons	↓ alcohol craving ↓ daily alcohol consumption ↓ neural cue-reactivity No difference in continuous abstinence
Brief interventions	Behavioral modification	↓ self-reported daily alcohol consumption ↓ self-reported heavy drinking episodes
CBT	Behavioral modification	↓ consumption frequency and quantity Not superior compared to MI and CM
AA	Behavioral modification	↑ abstinent days Cost effective Superior compared to CBT
Contingency management	Behavioral modification	↓ self-reported daily alcohol consumption ↓ self-reported heavy drinking days ↑abstinent days
Third-wave therapies	Behavioral modification	↓ self-reported daily alcohol consumption ↓ self-reported past week alcohol consumption
